# NCC regulation by WNK signal cascade

**DOI:** 10.3389/fphys.2022.1081261

**Published:** 2023-01-04

**Authors:** Shinichi Uchida, Takayasu Mori, Koichiro Susa, Eisei Sohara

**Affiliations:** Department of Nephrology, Graduate School of Medical and Dental Sciences, Tokyo Medical and Dental University, Tokyo, Japan

**Keywords:** pseudohypoaldosteronism type II, WNK kinases, OSR1, SPAK, NCC, KLHL3, CUL3

## Abstract

With-no-lysine (K) (WNK) kinases have been identified as the causal genes for pseudohypoaldosteronism type II (PHAII), a rare hereditary hypertension condition characterized by hyperkalemia, hyperchloremic metabolic acidosis, and thiazide-hypersensitivity. We thought that clarifying the link between WNK and NaCl cotransporter (NCC) would bring us new mechanism(s) of NCC regulation. For the first time, we were able to produce a knock-in mouse model of PHAII and anti-phosphorylated NCC antibodies against the putative NCC phosphorylation sites and discover that constitutive activation of NCC and increased phosphorylation of NCC are the primary pathogenesis of the disease *in vivo*. We have since demonstrated that this regulatory mechanism is mediated by the kinases oxidative stress-response protein 1 (OSR1) and STE20/SPS1-related proline/alanine-rich kinase (SPAK) (WNK–OSR1/SPAK-NCC signaling cascade) and that the signaling is not only important in the pathological condition of PHAII but also plays a crucial physiological role in the regulation of NCC.

## Pseudohypoaldosteronism type II

Worldwide, hypertension is very common, and even now, when there are many antihypertensive medications on the market, finding efficient treatment targets is still a pressing concern. Monogenic hypertensive conditions like Liddle syndrome and pseudohypoaldosteronism type II (PHAII) have helped us understand the physiological mechanisms that regulate blood pressure. PHAII was first described as familial hyperkalemic hypertension (FHHt) by Paver and Pauline (1964). In 1970, Gordon et al. reported that enhanced renal sodium reabsorption was the underlying cause of a 10-year-old girl’s hypertension, hyperkalemia, hyperchloremic metabolic acidosis, and suppression of renin-angiotensin systems (Gordon, 1986). Clinically, hyperkalemia is inevitable in patients in their teens due to impaired renal excretion of potassium, and hypertension develops in most cases thereafter. Aldosterone is suppressed and plasma renin activity is decreased, both of which indicate a tendency toward salt retention. However, due in part to high serum potassium, aldosterone may appear to be within the normal range. Administration of thiazide diuretics improves not only hypertension but also hyperkalemia and acidosis. Thiazide is beneficial in PHA II patients, and genetic anomalies of the NaCl cotransporter (NCC), the drug’s target molecule, have been speculated to be a potential contributing factor. However, no causative mutations had not been detected in the NCC gene.

## Discovery of WNK-OSR1/SPAK-NCC signaling cascade

**FIGURE 1:**
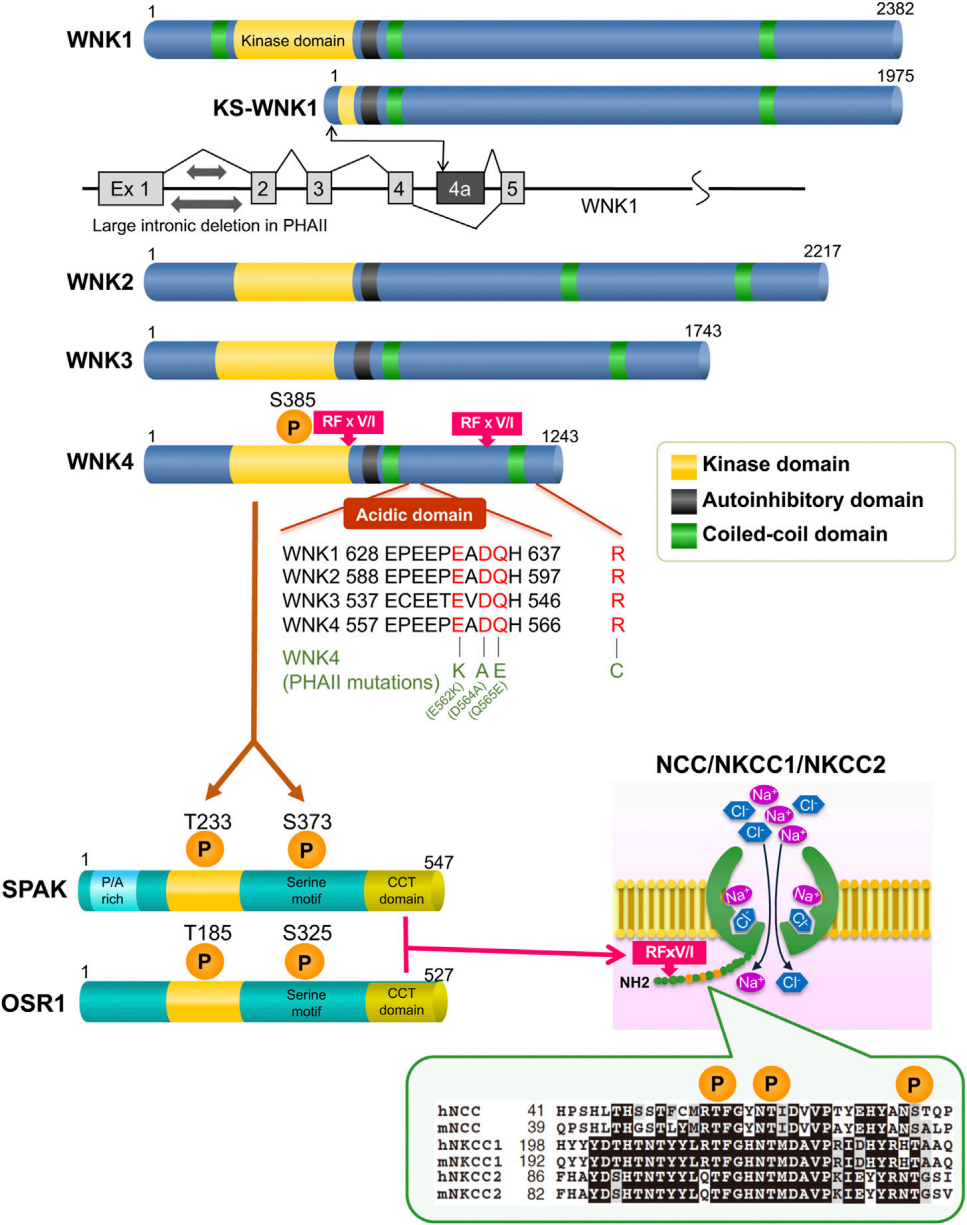
Overview of the WNK signaling molecules The CCT domain at the C-terminus of OSR1/SPAK is bound by the RFxV/I motif of the WNK kinases and SLC12A transporters. The PHAII mutations cluster in the region, which is the starting point for binding to KLHL3.

Since a conserved lysine (K) residue in the kinase’s catalytic domain is changed to a cysteine, WNK kinase, a member of the serine-threonine kinase family, earned its nickname (Xu et al., 2000). Presently, four types of WNK1 to four have been found in mammals, but there are no reports of WNK2 and WNK3 being aberrant in human kidney diseases. Full-length (long) WNK1 (L-WNK1) and kidney-specific WNK1 lacking the functional kinase domain are the two main isoforms of WNK1 that are known to exist (KS-WNK1). While KS-WNK1 was localized in the distal convoluted tubule (DCT), *in situ* hybridization demonstrated that the expression of L-WNK1 in the kidney was less than sensitive to detection (Delaloy et al., 2003). After a while, using a new method that uses “never-spliced” exon of WNK1 (in the case exon 8) as a reference, Vidal-Petiot et al. (2012) successfully quantified tissue-specific expression of WNK1 splice variants, including KS-WNK1. In particular, KS-WNK1 was revealed to be dominantly expressed in the DCT compared to L-WNK1. Cheng et al. isolated individual kidney tubule segments from KS-WNK1 KO mice tissue using the microdissection method and found that KS-WNK1 was most abundant in the DCT, followed by cortical thick ascending limb (cTAL), connecting tubule (CNT), and cortical collecting duct (CCD) (Cheng et al., 2013). In the study, the authors also demonstrated that KS-WNK1 stimulates ROMK-mediated K^+^ secretion and that KS-WNK1 plays a role in regulating Na^+^ transport in the CCD. Figure 1 illustrates the structure of WNK kinases. With two coiled-coil domains, an autoinhibitory domain with two phenylalanine residues, and a brief acidic domain, four WNK kinases are identical to one another in the kinase catalytic domain. The WNK1 mutation in PHAII was a large deletion of intron 1, as shown in Figure 1. In leukocytes from patients, a reverse transcription-PCR study revealed that WNK1 expression was elevated in PHAII patients (Wilson et al., 2001). A study from a French team utilizing WNK1 transgenic mice indicated that both isoforms are raised (Delaloy et al., 2008). Vidal-Petiot E et al. generated mice that recapitulated intron one deletion and observed that L-WNK1 was overexpressed only in the DCT and CNT and that the mice showed a PHAII phenotype (Vidal-Petiot et al., 2013). Chávez-Canales et al. crossed the WNK1–PHAII model mice [WNK1^(+/PHAII)^] lacking intron 1 with WNK4 knockout (KO) mice created afterward, and ectopic expression of L-WNK1 in the DCT and positive regulation of NCC independently of WNK4 were reported (Chávez-Canales et al., 2014). In KS-WNK1 transgenic mice, the total and phosphorylated forms of NCC and NKCC2 in renal cortex are reduced. These mice display renal Na^+^ wasting and lower blood pressure under normal Na^+^ diet (Liu et al., 2011). Conversely, KS-WNK1 knockout mice have increased expression of NCC and NKCC in renal cortex and hypertension (Hadchouel et al., 2010; Liu et al., 2011). These two studies are consistent with KS-WNK1 being an inhibitor of NCC in the DCT. On the other hand, WNK4 originally contained four missense mutations, three of which were located close to the coiled-coil domain in the protein’s first half. These variants were thought to alter the binding of WNK4 to other proteins, thereby changing its function as a kinase. Initially, many investigations (Kahle et al., 2003; Wilson et al., 2003; Yang et al., 2003; Yamauchi et al., 2004; Gamba, 2006; Garzón-Muvdi et al., 2007; Ring et al., 2007) were carried out to ascertain how WNK kinase regulates renal transporters. These studies were carried out in the form of co-overexpression of WNK1 or WNK4 and renal various transporters. However, it was unclear how exactly this control worked, particularly the intracellular signaling pathways involved. In 2006, Lalioti MD et al. used wild-type and PHAII mutant WNK4 genomic segments to create BAC transgenic mice. They discovered that mutant Tg mice [Tg(Wnk4^PHAII^)] have elevated blood pressure levels and hyperkalemic metabolic acidosis compared to wild-type mice, which is caused by increased NCC expression (Lalioti et al., 2006). In the study, NCC phosphorylation was not evaluated. Additionally, wild-type Tg mice [Tg(Wnk4^WT^)] suppress NCC and exhibit a Gitelman-like phenotype, which is inconsistent with the evidence that increased wild-type WNK4 due to reduced degradation by the KLHL3 mutation induces PHAII (Ohta et al., 2013; Shibata et al., 2013; Wakabayashi et al., 2013) rather than Gitelman syndrome. Although the *in vivo* study is important, it should be noted that forced expression systems, similar to previous *in vitro* studies, may not always accurately capture the physiological phenomenon. This is likely because of a lack of downstream signals or excessive modifications. To further comprehend the pathophysiology, we created Wnk4^D561A/+^ knock-in mice that carried the identical variation (D564A) as in human PHAII. The mice were verified to be human PHAII model mice with a dominant mode of inheritance because they displayed hyperkalemia, metabolic acidosis, and hypertension despite following a regular diet. All of these symptoms were alleviated by the administration of thiazide (Yang et al., 2007). Before this, the serine-threonine kinases oxidative stress-response protein 1 (OSR1) and STE20/SPS1-related proline/alanine-rich kinase (SPAK), which are members of the Ste20 kinase subfamily, were discovered as physiological substrates of WNKs (Moriguchi et al., 2005) (Figure 1). To examine the molecular pathogenesis of the disease in this mouse model, we first focused on NCC. Pacheco-Alvarez et al. discovered three key phosphorylation sites in NCC (T53, T58, and S71) involved in the activation based on the comparison with the NKCC1 sequence in a study using *Xenopus laevis* oocytes (Pacheco-Alvarez et al., 2006). With reference to the study, we created anti-phosphorylated NCC (pNCC) antibodies that detected probable Ser and Thr phosphorylation sites by OSR1 and SPAK. By employing anti-pNCC antibodies and demonstrating that pNCC was concentrated in apical plasma membranes in the DCT, Yang et al. (2007) demonstrated that NCC phosphorylation was dramatically elevated in the kidneys of PHAII model mice. We showed that WNK4 activates OSR1/SPAK, which in turn phosphorylates and activates NCC (WNK-OSR1/SPAK-NCC cascade) (Yang et al., 2007). Richardson et al. also reported that WNK1 phosphorylates and activates the three aforementioned NCC activation sites *via* OSR1/SPAK in *in vitro* experiments (Richardson et al., 2008). After that, we generated triple knock-in mice by mating WNK4^D561A/+^ knock-in mice with knock-in mice lacking OSR1 and SPAK kinase activities. This demonstrated that the activity of OSR1 and SPAK is required for the phosphorylation of NCC in the kidney (Chiga et al., 2011). The fact has also been shown in knockout mice of SPAK and OSR1, respectively (Yang et al., 2010; Lin et al., 2011). The enhanced NaCl reabsorption in the WNK4^D561A/+^ knock-in mice was mediated by phosphorylated and activated NCC, which was discovered to be the root of salt-sensitive hypertension. Additionally, because activated NCC in the DCT reabsorbs a significant amount of sodium, hyperkalemia is brought on by a reduction in sodium influx from downstream epithelial sodium channels (ENaC) and a reduction in K^+^ secretion through K channels (ROMK), which are functionally dependent on ENaC. WNK4 is expressed not only in the DCT cells but also in β−intercalated cells of the CCD (Kahle et al., 2004). β−intercalated cells exchange intracellular bicarbonate for external chloride through pendrin (SLC26A4), and therefore, account for renal base excretion. At the same time, these cells can also mediate thiazide-sensitive sodium chloride absorption when the pendrin-dependent apical chloride influx is coupled to apical sodium influx by the sodium-driven chloride/bicarbonate exchanger (NDCBE/SLC4A8) (Leviel et al., 2010). Pendrin activity was confirmed to be markedly increased in a mouse model carrying a WNK4 missense mutation (Q562E) (López-Cayuqueo et al., 2018), which may contribute to an increase in thiazide-sensitive sodium chloride absorption in CCD, and also to the development of metabolic acidosis in PHAII.

In addition, we discovered that phosphorylation of NCC attenuates its ubiquitination and was involved in NCC abundance at the apical membrane. Actually, in PHAII model mice, ubiquitination was reduced, which might be a factor in the overexpression of NCC at the membrane (Hossain Khan et al., 2012).

## Physiological significance of WNK-NCC signaling cascade

**FIGURE 2:**
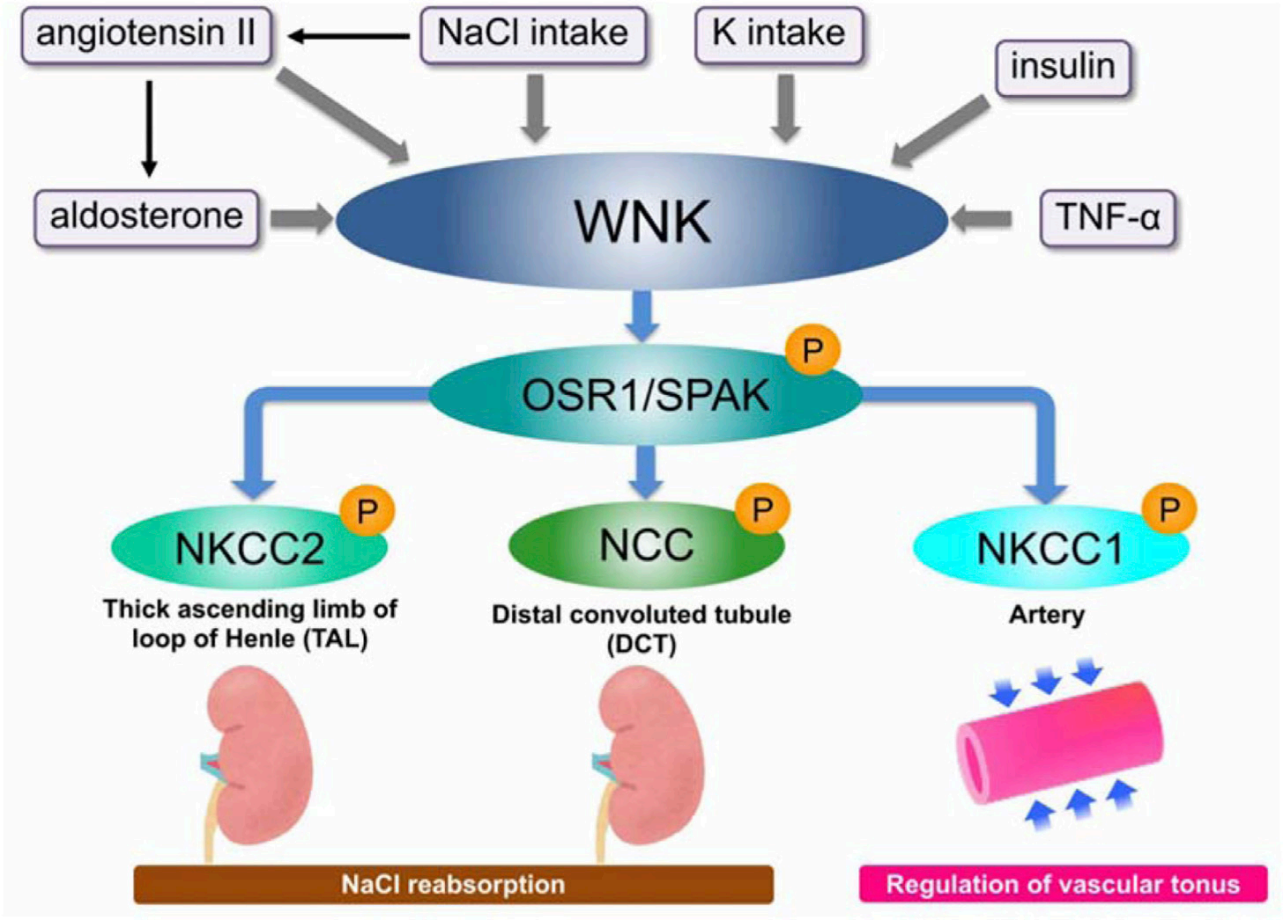
Overview of WNK signaling and its regulators WNK kinase forms a WNK–OSR1/SPAK–SLC12A signal that promotes phosphorylation and activation of downstream substrates, OSR/SPAK, and then NKCC1/2 and NCC. The signal is positively regulated by various regulatory factors such as aldosterone, angiotensin, and insulin.

By examining the variations in phosphorylation in normal and knock-in mice fed a high-salt or low-salt diet, it was further shown that WNK-OSR1/SPAK-NCC creates a phosphorylation cascade and plays a significant role in controlling renal electrolyte homeostasis. In wild-type mice, the cascade including NCC phosphorylation is suppressed by a high-salt diet and activated by a low-salt diet. In other words, it was initially controlled by the amount of salt consumed. However, in knock-in mice, the system remains constantly activated and is not inhibited by a high-salt diet. This is what is causing PHAII to continue to rise in salt reabsorption. When this system is suppressed by a high-salt diet, an additional exogenous aldosterone treatment can activate it. The activated state at low salt is suppressed by spironolactone, revealing aldosterone to be an upstream regulator of the cascade (Chiga et al., 2008; Vallon et al., 2009). This mechanism has been demonstrated to be an aldosterone effector system in the kidney in addition to the traditional aldosterone signaling pathway mediated by epithelial sodium channels (ENaC) (Figure 2). Castañeda-Bueno et al. created WNK4 knockout (KO) mice in 2012 and reported that they exhibit a Gitelman-like phenotype and that positive regulation of angiotensin II to NCC is mediated by WNK4 (Castañeda-Bueno et al., 2012). We also generated WNK4 KO mice and observed the marked reduction of phosphorylated and total NCC levels with the reduction of SPAK phosphorylation in the KO mice, demonstrating that WNK4 is the primary WNK positively regulating NCC (Takahashi et al., 2014). SPAK is also one of the most notable causal genes of essential hypertension because a genome-wide association study in the general hypertensive population revealed a substantial link with SNPs in the intronic region of SPAK (Wang et al., 2009). One of these flag SNPs (rs3754777) was knocked into HEK293T cells using a CRISPR/Cas system, which was revealed to cause increased protein expression and phosphorylation of SPAK. Additionally, the downstream Na-K-2Cl cotransporter-1 (NKCC1) was shown to be phosphorylated and activated, providing further evidence that the SNP is a functional polymorphism (Mandai et al., 2015).

## Inappropriate overactivation of NCC by WNK signal leads to salt-sensitive hypertension

The WNK signal is crucial for controlling blood pressure and maintaining NaCl homeostasis, in addition to being relevant for rare hereditary illnesses, according to the discovery of the WNK-OSR1/SPAK-NCC phosphorylation cascade. As previously indicated, excessive WNK-SPAK/OSR1-NCC phosphorylation signaling causes NCC phosphorylation to be improperly inhibited by salt consumption and salt to be improperly expelled in the urine, which causes salt retention and salt-sensitive hypertension. In other words, the discovery of the physiological activators of WNK signaling will provide insight into the new mechanisms of salt-sensitive hypertension. As a result, research has been done to find physiological activators of WNK signaling.

## Discovery of KLHL3 and CUL3, and their involvement in the pathophysiology of NCC activation

**FIGURE 3:**
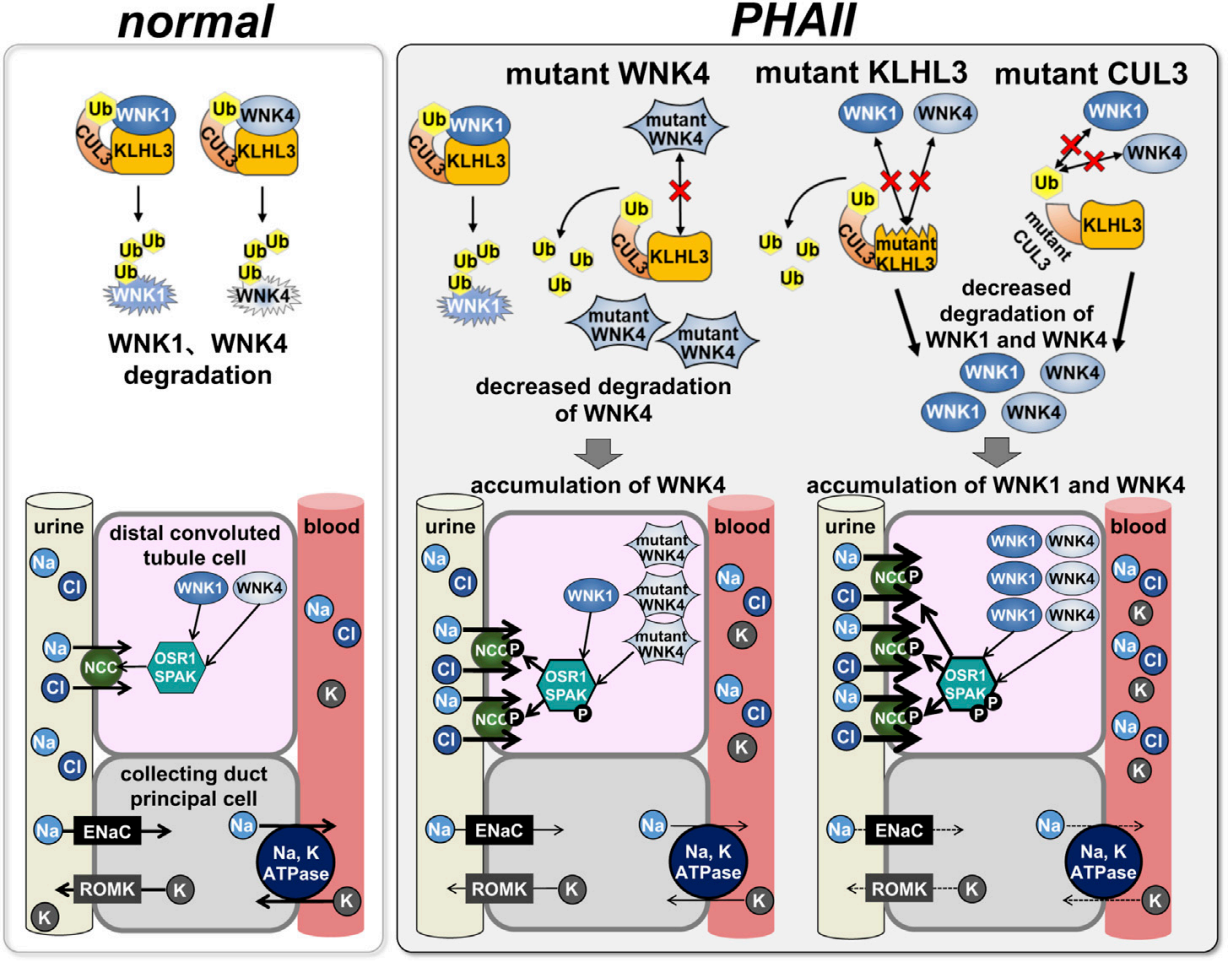
Effects of PHAII mutations in WNK signaling molecules on the signaling In normal states, CUL3, KLHL3, and WNK1/4 form the E3 ubiquitin ligase complex to regulate WNK1/4 degradation by the ubiquitin-proteasome system (left: normal); in PHAII, mutations in each intermolecular binding site fail this complex formation, subsequently, accumulation of WNK1/4 leads to activation of downstream OSR1/SPAK-NCC signaling.

While WNKs were found to activate NCC *via* OSR1/SPAK, the mechanism by which WNK mutations activate this cascade remained unclear. In 2012, two studies using next-generation sequencing analysis of PHAII pedigrees revealed that CUL3 and KLHL3 are new PHAII causal genes (Boyden et al., 2012; Louis-Dit-Picard et al., 2012). CUL3 and KLHL3 are part of the ubiquitin ligase complex that regulates protein degradation. Key elements of the ubiquitin or proteasome system that transfer ubiquitin moieties to substrates are ubiquitin ligases sometimes referred to as E3 ligases. CUL3 is a member of the Cullin family and binds to several substrate adapter proteins with BTB domains (Kipreos et al., 1996). Numerous members of the Kelch-like protein family are known, and they are substrate adapter proteins with five to seven Kelch domains that connect with substrates and a BTB domain that binds to CUL3 (Adams et al., 2000). KLHL–CUL3 E3 ligase substrates are diverse and be involved in many cellular functions. Based on these results, we postulated that the CUL3 and KLHL3 ubiquitin ligase complex would engage with any component of the WNK-OSR1/SPAK-NCC cascade to ubiquitinate and destroy it. First, we confirmed that KLHL3 interacts with CUL3 and WNK4 *in vitro*, induces WNK4 ubiquitination, and reduces WNK4 protein levels (Wakabayashi et al., 2013) (Figure 3). WNK4 ubiquitination was decreased and WNK4 protein levels were elevated as a result of decreased interaction between KLHL3 and WNK4 caused by PHAII-causing mutations in either protein. Furthermore, transgenic mice overexpressing WNK4 showed a PHAII phenotype, and Wnk4^D561A/+^ PHAII model mice had increased WNK4 protein (Wakabayashi et al., 2013). At about the same time as our report, Shibata et al. demonstrated that CUL3–RING ligases containing KLHL3 target ubiquitination of WNK4 and thereby regulate WNK4 levels, which in turn regulate levels of ROMK (Shibata et al., 2013). The KLHL3–CUL3 ligase complex’s role in WNK regulation was largely similar to our report in Wakabayashi et al., but this study concentrated primarily on ROMK as a result of WNK signaling. Ohta et al. (2013) also investigated WNK1’s *in vitro* interactions with KLHL3 and CUL3. The wild-type KLHL3–CUL3 E3 ligase complex ubiquitinated WNK1, whereas the mutant KLHL3–CUL3 E3 complex was shown to inhibit ubiquitination. CUL3 was knocked down using siRNA, which led to an increase in WNK1 protein levels and kinase activity in HeLa cells.

Additionally, we created and examined KLHL3^R528H/+^ knock-in mice, an ideal mouse model for KLHL3 PHAII, to further our understanding of the pathophysiology of PHAII induced by KLHL3 mutations (Susa et al., 2014). KLHL3^R528H/+^ knock-in mice exhibited salt-sensitive hypertension, hyperkalemia, and metabolic acidosis. It is interesting to note that the protein levels of WNK1 and WNK4 were both markedly higher in the kidney of KLHL3^R528H/+^ mice, suggesting that mutant KLHL3 kinase expedited the phosphorylation of the WNK-OSR1/SPAK-NCC cascade in mice. Fluorescence correlation spectroscopy was used to evaluate the binding of TAMRA-labeled WNK1 and WNK4 peptides to full-length KLHL3 to determine whether mutant KLHL3 R528H can interact with WNK kinase. The results showed that neither WNK1 nor WNK4 could bind to KLHL3 R528H (Susa et al., 2014).

That KLHL3-CUL3 complex binds to and interacts with WNK1 and WNK4, which causes their ubiquitination and lowers their protein levels, according to numerous *in vitro* and *in vivo* studies. The combination of CUL3 and KLHL3 binds WNK kinases and modestly degrades them to control the activity of the WNK-OSR1/SPAK-NCC cascade for sodium homeostasis under normal physiological settings. However, mutations in these molecules decrease their ubiquitination activity and prevent them from attaching. Slower degradation causes WNK kinases to accumulate (Figure 3). Recently, a whole-exome analysis of a family with a PHAII phenotype and a dominant inheritance form revealed a new missense mutation (p.Asp635Glu) in exon seven of WNK1 (Louis-Dit-Picard et al., 2020). Functional studies on *Xenopus laevis* oocytes and HEK293T cells showed that this mutation strongly reduces KS-WNK1 ubiquitination by the KLHL3–CUL3 complex rather than the L-WNK1 isoform. This mutation is on the short conserved acidic motif, the binding motif for the KLHL3–CUL3 complex. Knock-in mice with the same mutation displayed the PHAII phenotype. It was proposed that the mechanism of KS-WNK1 increase is an important pathophysiology of WNK1-induced PHAII development (Louis-Dit-Picard et al., 2020).

As a result, permanent overactivation of the WNK–OSR1/SPAK-NCC cascade promotes NCC-mediated salt reabsorption. There was continuing discussion over whether WNK4 was a positive or negative regulator of the NCC before the discovery of this WNK degradation pathway, and there was evidence to support both hypotheses. Chloride stabilizes the inactive conformation of WNK1, preventing kinase autophosphorylation and activation (Piala et al., 2014). Similarly, WNK4 can have different effects on NCC depending on the level of intracellular chloride (Bazúa-Valenti et al., 2015). Although there are numerous modifying factors in WNK signaling, our results from *in vivo* studies do not negate the negative effect of WNK on NCC, which was initially established by *in vitro* experiments with oocytes and cultured cells, as mentioned in the previous report (Takahashi et al., 2014). Our research on WNK-NCC signaling has significantly impacted the discussion about the physiological and pathological mechanisms that are prevalent *in vivo*.

## Insulin links WNK-NCC signal activation and salt-sensitive hypertension in metabolic syndrome

Clinical data showed that patients with obesity and metabolic syndrome had increased insulin resistance (Chen et al., 2009), sparking research into the underlying mechanisms. Metabolic syndrome is characterized by insulin resistance, leading to hyperinsulinemia. It has been shown that insulin increases salt sensitivity and activates the WNK-OSR1/SPAK-NCC signaling (Sohara et al., 2011; Komers et al., 2012; Chávez-Canales et al., 2013; Takahashi et al., 2014). A high-salt diet has little effect on WNK signaling in db/db animal models of the hyperinsulinemic metabolic syndrome (Nishida et al., 2012; Ishizawa et al., 2019). Furthermore, Nishida et al. showed that inhibiting PI-3K or Akt in db/db mice reduced elevated NCC phosphorylation, demonstrating that insulin increases WNK-OSR1/SPAK-NCC signaling *via* the PI-3K/Akt pathway. Indeed, KLHL3 is phosphorylated at S433 by Akt, resulting in defective degradation of WNK kinase, due to decreased binding between WNKs and KLHL3 (Yoshizaki et al., 2015). These findings suggest that WNK signaling is a promising target for metabolic syndrome’s blood pressure management.

## Calcineurin inhibitor activates the WNK-NCC axis, leading to PHAII-like phenotype

Both physiologically and therapeutically, the effects of calcineurin (CaN) and its inhibitors on WNK signaling are crucial. Cyclosporine is known to cause hypertension and hyperkalemia as side effects. These adverse consequences resemble the PHAII phenotype, which raises the possibility that calcineurin inhibitors could stimulate the WNK-NCC signal. Farfel’s team observed that CaN inhibitor-fed rats developed salt-sensitive hypertension and that their kidneys showed much higher levels of WNK4 protein and NCC phosphorylation (Melnikov et al., 2011). Ellison’s team extended these findings to people by demonstrating that kidney transplant recipients who had received tacrolimus had fractional chloride excretion in response to bendroflumethiazide, an NCC inhibitor, than those who had not received tacrolimus; as well as higher renal NCC abundance (Hoorn et al., 2011). These findings may help to partially explain how calcineurin inhibitors-cause hypertension and hyperkalemia, and they also imply that tacrolimus patients had higher levels of cheap NCC abundance. It demonstrates that well-tolerated thiazide diuretics may be particularly effective in preventing the complications of CNI treatment. Additionally, it has been proposed that NCC is directly dephosphorylated by calcineurin. Shoda et al. demonstrated that tacrolimus, a CaN inhibitor, inhibits the fast dephosphorylates of NCC after oral potassium treatment, irrespective of OSR1/SPAK (Shoda et al., 2017). This suggests that high potassium CaN is involved in NCC dephosphorylation.

## Tumor necrosis factor (TNF) α activates the WNK1-NCC signal, leading to salt-sensitive hypertension in chronic kidney disease (CKD)

The most frequent comorbidity linked to CKD is hypertension (Townsend and Taler, 2015). One of the main reasons for the development of hypertension in CKD patients is increased salt sensitivity. Recent research reveals that insufficient sodium processing in the tubules, in addition to decreased glomerular filtration rate, causes salt sensitivity in CKD (Gonzalez-Villalobos et al., 2013; Kobayashi et al., 2017). Additionally, a recent publication to the NEJM stating that thiazides, which are NCC inhibitors, are successful in treating advanced CKD raises the possibility that NCC is responsible for this elevated salt sensitivity in CKD (Agarwal et al., 2021).

On the other hand, studies are emerging suggesting that the immune system is important in hypertension (Norlander et al., 2018; Rucker et al., 2018). According to these findings, the immune system may be involved in the excessively high renal salt retention that results in hypertension. Studies in which reduction or suppression of TNF inhibited the development of hypertension in mice in response to Ang II infusion, for instance, suggested that the inflammatory cytokine TNFα is involved in hypertension (Guzik et al., 2007; Zhang et al., 2014). Furthermore, clinically, TNFα antagonists were reported to have antihypertensive effects in patients with rheumatoid arthritis (Yoshida et al., 2014). Activating the WNK1-SPAK-NCC phosphorylation cascade and causing salt-sensitive hypertension in CKD animal models are two recent findings made by Furusho et al. (2020). Renal TNFα increased WNK1 protein expression by suppressing NEDD4-2, another E3 ligase that degrades WNK1. Thus, in CKD kidneys with elevated renal TNFα, the WNK1-SPAK-NCC signaling pathway is activated, resulting in salt-sensitive hypertension. These findings suggest that the immune system controls the WNK phosphorylation pathway, which is connected to salt-sensitive hypertension in CKD.

## Challenges in drug discovery and biomarker development

Although there are thiazides that only control NCC, the following facts make it worthwhile to block signals from upper levels in addition to NCC. As previously indicated, WNK signaling is crucial in the control of sodium delivery *in vivo*, which is influenced by several regulatory factors. Physiologically, it may be a promising new drug target because it is involved not only in salt regulation but also in the control of vasoconstriction. WNK4 regulates adipocyte early differentiation and is expressed in adipose tissue. Mice lacking WNK4 demonstrated resistance to obesity from a high-fat diet (Takahashi et al., 2017). Additionally, SPAK is expressed in white adipose tissue, and SPAK kinase-inactivated knock-in mice, in which the WNK phosphorylation activation site was inactivated, displayed resistance to obesity and hepatic steatosis brought on by high-fat diets (Torre-Villalvazo et al., 2018). Additionally, it has been suggested that a lack of SPAK enhances the intestine’s innate immune homeostasis, which is crucial for regulating or reducing pathogenic responses in inflammatory bowel illnesses (Zhang et al., 2013). These results imply that WNK signaling inhibitors may be new antihypertensive diuretics and metabolic ameliorators. Fluorescence correlation spectroscopy was used to carry out high-throughput screening of WNK-SPAK binding inhibitors (Mori et al., 2013) (Figure 1). Then, using ELISA, a screening of direct SPAK inhibitors was carried out, which made it possible to find several intriguing compounds that had *in vivo* WNK signaling inhibitory action (Kikuchi et al., 2015). A recent *in silico* structural analysis of one of the candidates, STOCK1S-14279, revealed that it binds to an allosteric pocket in the conserved carboxy-terminal (CCT) domain of SPAK and inhibits its interaction with WNK. This candidate has been cited and used in subsequent studies as a SPAK inhibitor (Zhang et al., 2020; Jonniya et al., 2021). Furthermore, we established a quantitative detection method for urinary phosphorylated NCC by ELISA as a biomarker for the evaluation of WNK signaling activation (Isobe et al., 2013). Gitelman syndrome and PHAII patients are two hereditary renal diseases that can change NCC activity, and the test is less invasive and useful for evaluating WNK signaling activity as well as for screening for these conditions. A marked decrease in Gitelman patients and a marked increase in PHAII patients’ NCC activity were noted (Isobe et al., 2013).

The discovery of the WNK signaling, a specific and potent regulator of NCC, has provided many new insights into NCC regulation. Our knowledge of Na, Cl, and K handling in the kidney has advanced along with our awareness of the role of NCC in physiological and pathological aspects. We are greatly looking forward to the development of signal transduction research in this field.
